# Diagnosis and treatment of intramedullary osteosclerosis: a report of three cases and literature review

**DOI:** 10.1186/s12891-020-03758-5

**Published:** 2020-11-19

**Authors:** Kensaku Abe, Norio Yamamoto, Katsuhiro Hayashi, Akihiko Takeuchi, Shinji Miwa, Kentaro Igarashi, Takashi Higuchi, Yuta Taniguchi, Hirotaka Yonezawa, Yoshihiro Araki, Sei Morinaga, Yohei Asano, Hiroyuki Tsuchiya

**Affiliations:** Department of Orthopaedic Surgery, Graduate School of Medical Sciences, Kanazawa University, 13-1 Takara-machi, Kanazawa-shi, Ishikawa-ken 920-8641 Japan

**Keywords:** Intramedullary osteosclerosis, 99mTc- methylene diphosphonate (MDP) triphasic bone scan, Open biopsy

## Abstract

**Background:**

Intramedullary osteosclerosis (IMOS) is a rare condition without specific radiological findings except for the osteosclerotic lesion and is not associated with family history and infection, trauma, or systemic illness. Although the diagnosis of IMOS is confirmed after excluding other osteosclerotic lesions, IMOS is not well known because of its rarity and no specific feature. Therefore, these situations might result in delayed diagnosis. Hence, this case report aimed to investigate three cases of IMOS and discuss imaging findings and clinical outcomes.

**Case presentation:**

All three cases were examined between 2015 and 2019. The location of osteosclerotic lesions were femoral diaphyses in the 60-year-old man (Case 1) and 41-year-old woman (Case 2) and tibial diaphysis in the 44-year-old woman (Case 3). All cases complained of severe pain and showed massive diaphyseal osteosclerotic lesions in plain radiograms and computed tomography (CT) scans. Cases 2 and 3 were examined using the triphasic bone scan, and a fusiform-shaped intense area of the tracer uptake on delayed bone image was detected in both cases without (Case 2) or slightly increased vascularity (Case 3) on the blood pool image, which was reported as a specific finding of IMOS. Open biopsy was performed in all cases, and histologic section showed trabecular bone sclerosis with hypocellular fibrous tissues, finally diagnosed as IMOS. The pain was sharply improved after biopsy and kept at the latest follow-up periods (34, 33, and 6 months in Cases 1, 2, and 3, respectively).

**Conclusions:**

Massive sclerotic lesions with severe pain in the diaphyseal region of long bones should be considered as IMOS to avoid the delayed diagnosis, although other sclerotic bony lesions should be carefully excluded. Triphasic bone scan with a fusiform-shaped intense area of tracer uptake on delayed bone image and without or slightly increased vascularity on the blood pool image will help confirm IMOS. The role of open biopsy was to confirm the diagnosis of IMOS and to give the severe pain relief immediately in the three cases, although more cases and long-term follow-up are necessary.

## Background

Sclerotic intramedullary lesions on radiography in patients with pain in the long bones have several differential considerations, such as malignancy (e.g., osteosarcoma, lymphoma or metastasis), inflammation (e.g., chronic osteomyelitis or chronic recurrent multifocal osteomyelitis), trauma (e.g., healing stress fracture), and bone dysplasia [1, 2]. Intramedullary osteosclerosis (IMOS) was first reported by Abdul-Karim FM in 1988 as an unusual pathological condition characterized by endosteal new bone formation and sclerosis in the diaphysis of long bones in adults [3]. IMOS is a rare condition that is not associated with family history and infection, trauma, or systemic illness [4]. Moreover, the diagnostic criteria and treatment strategy have not yet been established. So, those situations might result in the delay of diagnosis. Whole-body bone scan usually shows the tracer uptake on the certain sclerotic lesions, however, a fusiform-shaped intense area of tracer uptake on delayed bone image in triphasic bone scan was reported as the possibility of specific findings in IMOS [1]. Here, we present three cases of IMOS which were examined between 2015 and 2019, and the purpose of this report is to evaluate the imaging findings including the triphasic bone scan, the role of biopsy and clinical outcome with a literature review. Written informed consent was obtained from all patients for the publication of this case report and any accompanying images.

## Case presentation

### Case 1

**Fig. 1:**
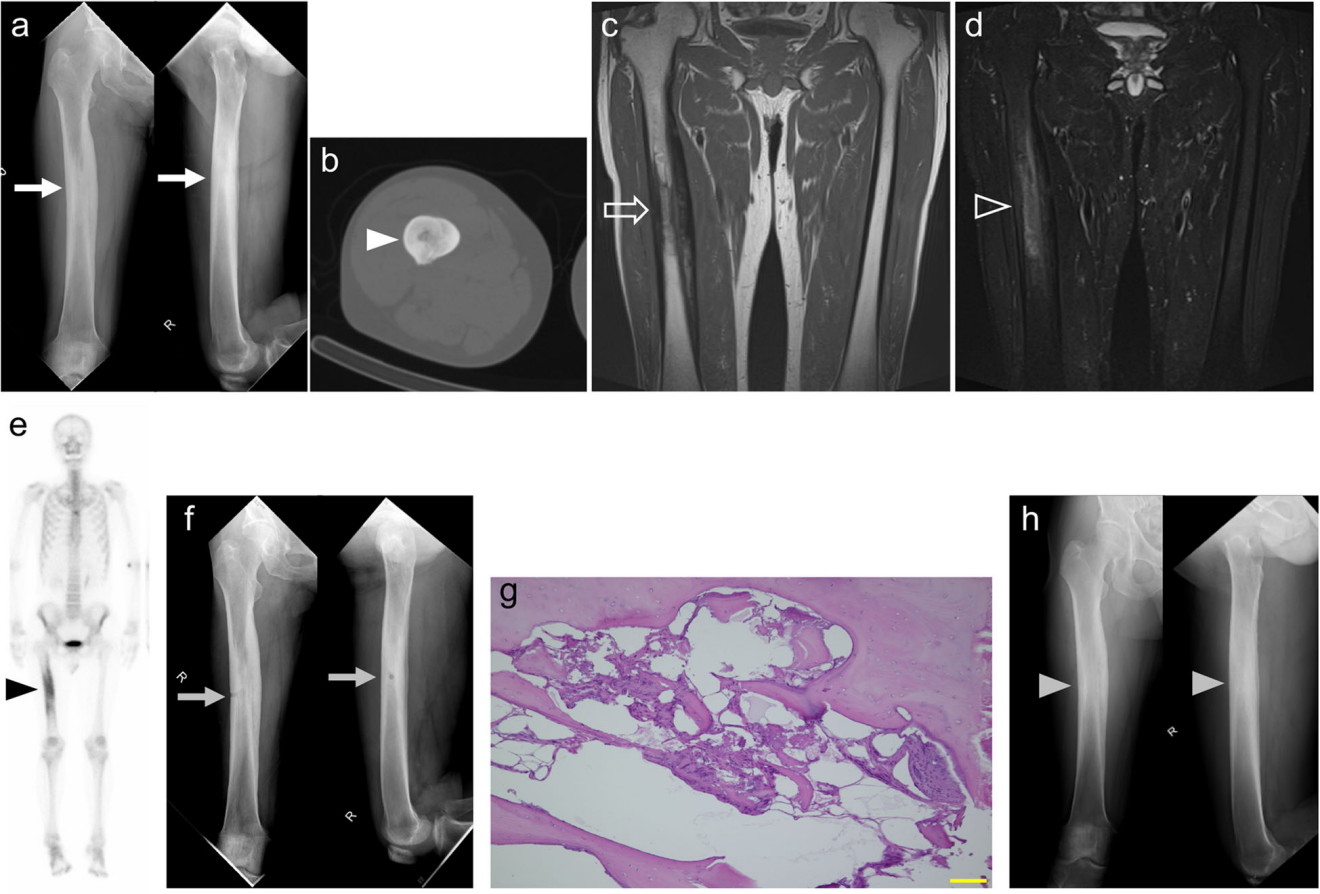
Case 1. A 60-year-old man with intramedullary osteosclerosis of the right femur. Preoperative radiography shows massive sclerotic intramedullary lesion of the right femoral shaft and cortical bone thickening (white arrow). Left panel is an anteroposterior view. Right panel is lateral view (**a**). Preoperative CT (axial) shows medullary cavity narrowing (white arrowhead) (**b**). Preoperative MRI of T1-weighted SE sequence was hypointense, corresponding to the intramedullary sclerosis visualized on radiograph and CT (whiteline arrow) (**c**), and T2-weighted STIR sequence showed high signal intensity in the medullary cavity and without soft tissue mass (whiteline arrowhead) (**d**). Whole-body 99mTc-MDP bone scan showed abnormal tracer uptake in the right femur (black arrowhead) (**e**). Postoperative radiography. Gray arrow indicates the biopsy hole (**f**). Hematoxylin-eosin staining of the specimen from an open biopsy showed trabecular bone sclerosis with hypocellular fibrous tissue. Scale bar indicates 100 μm (**g**). Radiography at 39 months postoperatively. The bone hole was completely repaired (gray arrowhead) (**h**)

A 60-year-old man had a severe pain on the right thigh that worsened gradually, and nonsteroidal anti-inflammatory drugs (NSAIDs) were ineffective. He had only severe spontaneous pain and did not have local heat, swelling, or redness. He had no notable medical and family history. Laboratory data including inflammatory reaction were within normal limits. Radiography (Fig. 1a) and computed tomography (CT) (Fig. 1b) showed massive sclerotic intramedullary lesion of the right femoral shaft, cortical bone thickening, and narrowing of the medullary cavity. T1-weighted spin-echo (SE) sequence (echo time [TE], 10 msec; repetition time [TR], 550 msec; and slice thickness, 4 mm) of magnetic resonance imaging (MRI) was hypointense corresponding to the intramedullary sclerosis visualized on radiograph and CT (Fig. 1c) and T2-weighted short inversion time inversion recovery (STIR) sequence (TE, 60 msec; TR, 6000 msec; and slice thickness, 4 mm) showed high signal intensity in the medullary cavity. There was no soft tissue mass (Fig. 1d). Whole-body 99mTc-methylene diphosphonate (MDP) bone scan showed abnormal tracer uptake with intense sclerosis in the intramedullary region (Fig. 1e). Then, open biopsy was carried out under fluoroscopic guidance (Fig. 1f). The permanent histologic section showed trabecular bone sclerosis with hypocellular fibrous tissue. (Fig. 1g). The specimen culture was negative. The patient was finally diagnosed with IMOS based on the no specific imaging findings, and histological findings which was consistent with IMOS. The pain improved sharply postoperatively, and there was almost no pain after 6 months. However, the pain began to recur with the remodeling of the biopsy hole at 8 months postoperatively; the pain was managed by the administration of NSAIDs. After that, although the pain was intermittent and so was the treatment with NSAIDs, the quality of life was not compromised. At 39 months postoperatively, the plain radiography showed no enlargement of sclerotic lesion and a repair of biopsy hole (Fig. 1h). In this case, it took approximately 2 years from the onset of the symptom to the diagnosis.

### Case 2

**Fig. 2:**
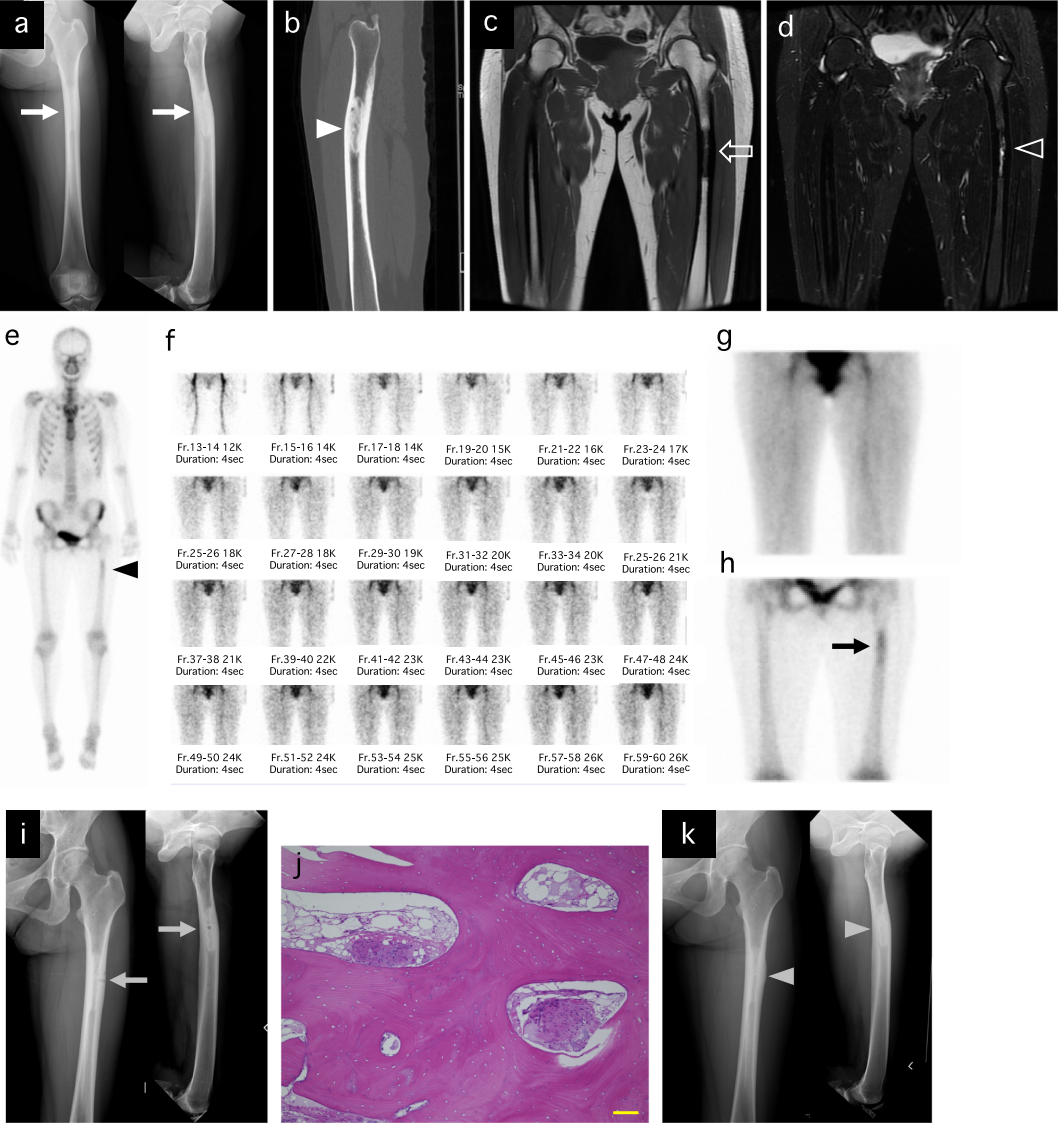
Case 2. A 41-year-old woman with intramedullary osteosclerosis of the left femur. Preoperative radiography shows a massive sclerotic intramedullary lesion of the right femoral shaft and cortical bone thickening (white arrow). Left panel is the anteroposterior view. Right panel is the lateral view (**a**). Preoperative CT (sagittal) shows medullary cavity narrowing (white arrowhead) (**b**). Preoperative MRI of T1-weighted SE sequence (coronal) was hypointense, corresponding to the intramedullary sclerosis visualized on radiograph and CT (whiteline arrow) (**c**) and T2-weighted STIR sequence (coronal) showed high signal intensity in the medullary cavity and without soft tissue mass (whiteline arrowhead) (**d**). Whole-body 99mTc-MDP bone scan showed an abnormal tracer uptake in the left femur (black arrowhead) (**e**). Triphasic bone scan (**f**, **g**, **h**). The initial vascular phase (**f**) and blood pool images (**g**) at 2 min showed no evidence of increased vascularity or soft tissue tracer pooling. Delayed bone images (**h**) showed a fusiform-shaped intense area of the tracer uptake in the left femur diaphysis (black arrow). Postoperative radiography. Gray arrow indicates the biopsy hole (**i**). Hematoxylin-eosin staining of the specimen from an open biopsy showed the thickened trabecular bone and fibrous hyperplasia with little inflammatory cell infiltration. Scale bar indicates 100 μm (**j**). Radiography at 39 months postoperatively. The bone hole was completely repaired (gray arrowhead) (**k**)

A 41-year-old woman had a severe pain on the left thigh. She had taken NSAIDs for a few months; however, the intermittent pain persisted. She had no notable medical and family history. She had only severe spontaneous pain and did not have local heat, swelling, or redness. No abnormal laboratory results including inflammatory reaction were observed. Radiography (Fig. 2a) and CT (Fig. 2b) showed a sclerotic intramedullary lesion of the left femoral shaft and narrowing of the medullary cavity. T1-weighted SE sequence (TE, 13 msec; TR, 483 msec; and slice thickness, 4 mm) of MRI (Fig. 2c) showed hypointense corresponding to the intramedullary sclerosis visualized on radiograph and CT, and T2-weighted STIR sequence (TE, 68 msec; TR, 4200 msec; and slice thickness, 4 mm) (Fig. 2d) showed high signal intensity in the medullary cavity and absence of soft tissue mass. These findings were incompatible with bone tumors and stress fracture. Whole-body bone scan revealed abnormal tracer uptake in the left femur (Fig. 2e). Moreover, triphasic bone scan was performed. The initial vascular phase (Fig. 2f) and blood pool images (Fig. 2g) at 2 min showed no evidence of increased vascularity or soft tissue tracer pooling. Delayed bone images (Fig. 2h) showed a fusiform-shaped intense area of tracer uptake in the diaphysis of the left femur. These findings were not consistent with osteomyelitis. Open biopsy was then performed under fluoroscopic guidance (Fig. 2i), and the histological findings showed a thickened trabecular bone and fibrous hyperplasia with slight inflammatory cell infiltration. (Fig. 2j). The specimen culture was negative. This patient was finally diagnosed with IMOS after the exclusion of other diseases based on the imaging and histological findings. The pain improved sharply postoperatively and was relieved 3 months postoperatively. At 33 months postoperatively, the plain radiography showed no enlargement of sclerotic lesion and the repaired of biopsy hole (Fig. 2k). And, the pain did not recur at the latest follow-up. In this case, it took approximately 8 months from onset of the symptom to the diagnosis.

### Case 3

**Fig. 3:**
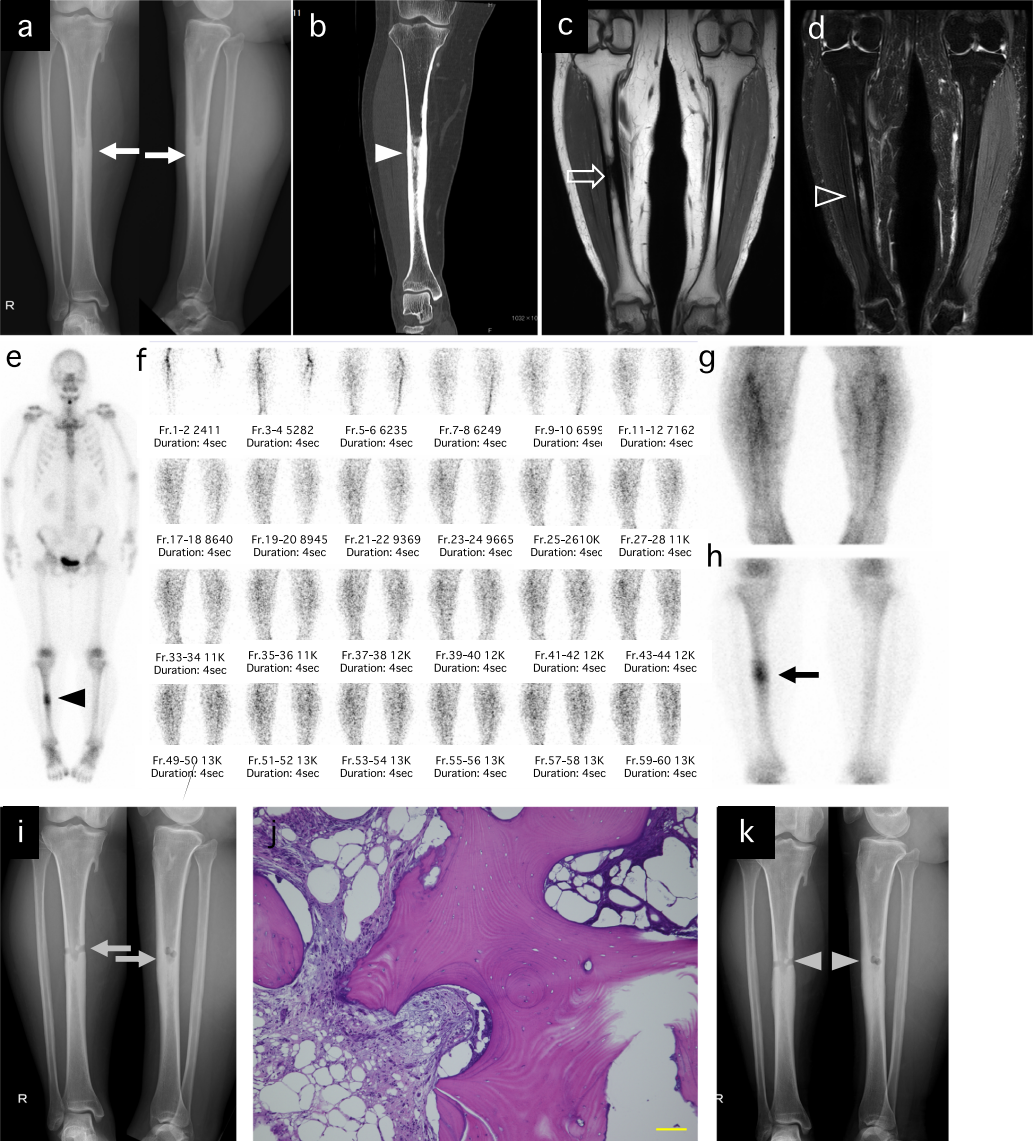
Case 3. A 44-year-old woman with intramedullary osteosclerosis of the right tibia. Preoperative radiography shows massive sclerotic intramedullary lesion of the right tibial shaft and cortical bone thickening (white arrow). Left panel is the anteroposterior view. Right panel is the lateral view (**a**). Preoperative CT (coronal) shows medullary cavity narrowing (white arrowhead) (**b**). Preoperative MRI of T1-weighted SE sequence was hypointense, corresponding to the intramedullary sclerosis visualized on radiograph and CT (whiteline arrow) (**c**) and fat-suppressed T2-weighted sequence showed high-signal intensity in the medullary cavity and without soft tissue mass (whiteline arrowhead) (**d**). Whole-body 99mTc-MDP bone scan showed an abnormal tracer uptake in the left femur (black arrowhead) (**e**). Triphasic bone scan (**f**, **g**, **h**). The initial vascular phase (**f**) and blood pool images (**g**) at 2 min showed no evidence of increased vascularity or soft tissue tracer pooling. Delayed bone images (**h**) showed a fusiform-shaped intense area of tracer uptake in the left femur diaphysis (black arrow). Postoperative radiography. Gray arrow indicates the biopsy hole (**i**). Hematoxylin-eosin staining of the specimen from an open biopsy. The cancellous bone was replaced by a new trabecular bone and hypocellular fibrous hyperplasia. Scale bar indicates 100 μm (**j**). Radiography at 6 months postoperatively showed a slightly repaired biopsy hole (gray arrow) (**k**)

A 44-year-old woman had a severe pain on the right lower thigh. She had used NSAIDs for a few months; however, the pain did not ameliorate. Then, she took opioids, and the pain was slightly relieved. She had no notable medical and family history. She had only severe spontaneous pain and did not have local heat, swelling, or redness. There were no abnormal laboratory results including inflammatory reaction. Radiography (Fig. 3a) and CT showed (Fig. 3b) a sclerotic intramedullary lesion on the right tibial shaft and narrowing of the medullary cavity. T1-weighted SE sequence (TE, 15 msec; TR, 450 msec; and slice thickness, 5 mm) of MRI (Fig. 3c) was hypointense, corresponding to intramedullary sclerosis visualized on radiograph and CT, and the fat-suppressed T2-weighted sequence (TE, 100 msec; TR, 3021 msec; and slice thickness, 5 mm) (Fig. 3d) showed high signal intensity in the medullary cavity and absence of soft tissue mass. Whole-body bone scan showed abnormal tracer uptake in the left femur (Fig. 3e). Moreover, triphasic bone scan was performed. The initial vascular phase (Fig. 3f) showed no evidence of increased vascularity or soft tissue tracer pooling, and blood pool images (Fig. 3g) at 2 min showed little evidence of increased vascularity. Delayed bone images (Fig. 3h) showed a fusiform-shaped intense area of tracer uptake in the diaphysis of the right tibia. Then, open biopsy was performed under fluoroscopic guidance (Fig. 3i), and the permanent histologic section showed that the cancellous bone was replaced by a new trabecular bone, and hypocellular fibrous hyperplasia. (Fig. 3j). The specimen culture was negative. The patient was finally diagnosed with IMOS after the exclusion of other diseases. The pain improved considerably postoperatively, then worsened and improved, and reduced to approximately one-third of the preoperative pain at 6 months postoperatively. She continues to use NSAIDs but has not needed opioids. At 6 months postoperatively, plain radiography showed the slight a slightly repaired biopsy hole (Fig. 2k). In this case, it took approximately 8 months from onset of the symptom to the diagnosis.

## Discussion and conclusions

IMOS is a rare benign pathologic condition of unknown etiology [2]. The essential findings in IMOS are adult-onset, female predominance, lack of a similar condition in family members, and asymmetric distribution without the extensive periosteal new bone formation and soft tissue involvement that affects a portion of the diaphysis of one or more long bones of one or both lower extremities [4, 5]. However, IMOS has been a diagnosis of exclusion [2, 4]. Due to its rarity and the lack of specific imaging and histological findings, it will require a long time from the onset to confirm the diagnosis. All three cases in this study took a long duration (2 years in case 1 and 8 months in cases 2 and 3) to reach the diagnosis of IMOS.

Laboratory results are typically normal [3, 4, 6]. In this case series, there were no notable findings in the laboratory data. Some studies suggested the absence of a hereditary pattern, female predominance, mechanical pain, and absence of periosteal hyperostosis favoring IMOS [4, 6]. However, several other studies suggest these differential points are not diagnostically useful in many cases [7–9]. Radiographically, the benign-appearing IMOS that develops without the extensive periosteal new bone formation and soft tissue abnormality, which are characteristic of idiopathic IMOS, is different from the typical findings of malignant tumors, such as osteosarcoma, lymphoma, and osteoblastic metastasis [4]. Although osteosarcoma, lymphoma, and osteoblastic metastasis in the diaphysis of a tubular bone have some characteristics that resemble those of IMOS, such as a sclerotic lesion, periosteal elevation and soft tissue extension are often evident [6, 9, 10]. As there was a sclerotic intramedullary lesion without the periosteal reaction and soft tissue extension in this case series, the malignant tumors could be excluded. MRI findings on fat-suppressed T2-weighted images varied from the absence of associated bone marrow edemalike signal intensity [11] to the slightly hyperintense bone marrow [2] in IMOS. Skiadas et al. reported that slightly hyperintense bone marrow was considered as a reaction process in IMOS [2]. The possibility of chronic sclerosing osteomyelitis usually can be discarded, owing to the absence of clinical and laboratory findings of infection [12]. In this case series, chronic sclerosing osteomyelitis could be excluded from clinical findings and laboratory data in spite of the bone marrow edemalike signal intensity.

Pathologically, The peripheral and major part of the lesion is characterized by the replacement of normal spongiosa by markedly sclerotic and thickened trabeculae that encompassed the marrow cavity with a variable degree of mineralization and maturity [4]. However, the exclusion of other osteosclerotic disease with imaging findings is indispensable for diagnosis of IMOS. Puranik et al. reported the usefulness of triphasic bone scan in their case report. They described that radiological features in skeletal dysplasia are not pathognomonic, and a triphasic bone scan helps narrow down the differential conditions [1]. However, there was no other report about the triphasic bone scan to diagnose IMOS. So, we conducted the triphasic bone scan in two cases (cases 2 and 3). Only delayed bone images showed an intense area of tracer uptake in case 2, and delayed bone images showed an intense area of tracer uptake, and blood pool images showed a slightly intense area of tracer uptake in case 3. These results of a severe intense area in delayed bone images and no or slightly intense area in the blood pool image coincide with those in the report by Puranik et al. Zhang reported that malignant bone tumor and osteomyelitis can all demonstrate three phase positivity (enhanced blood flow, soft tissue hyperemia, and increased uptake in delayed phase) [13]. The vascular phase dynamic images were found to have relatively poor sensitivity with stress fractures. The blood pool imaging was positive in the stress fractures, and especially lateral/medial blood pool imaging was significantly better than anterior/posterior images. The delayed phase imaging was also positive in the stress fractures [14]. According to these reports, inflammatory diseases such as osteomyelitis, progressive malignant tumors and stress fracture could be excluded. And then, it was consistent with IMOS that the diseases that strongly accumulated in the delayed phases were confined to the bones. These differential diagnoses were described in Table 1. However, further analysis of the usefulness of the triphasic bone scan is necessary.

**Table 1:** The differential diagnosis using triphasic bone scan

Phase	Malignancy [13]	Osteomyelitis [13]	Stress fracture [14]	IMOS [1], [our cases]
Phase 1: Vascular phase	Enhanced blood flow	Enhanced blood flow	Relatively poor sensitivity	Absent
Phase 2: Soft tissue (Blood pool)	Soft-tissue hyperemia	Soft-tissue hyperemia	Lateral/medial blood pool imaging was positive and significantly better than anterior/posterior images	Absent or slightly uptake
Phase 3: Delayed (bone)	Increased uptake	Increased uptake	Focal increased tracer uptake	Fusiform-shaped intense area of the tracer uptake

Other differential diseases with spontaneous pain and osteosclerotic change include osteoid osteoma. If these cases were osteoid osteoma, a nidus could be found CT and MRI [15]. We carefully checked both MRI and CT and there was no nidus in both imaging. So, in this case series, osteoid osteoma could be excluded.

Given the absence of known etiology but based on the belief that bone marrow edema causes pain, many authors have advocated pain relief in IMOS, whether by reaming, curettage, or making a window at the lesion [7, 16, 17]. Kang et al. treated their patient with IMOS with intramedullary reaming. Once, the pain had subsided. However, the pain redeveloped at the time of establishing the medullary canal. They described that medullary decompression appears to be important in pain relief, although it does not correct the primary cause of disease [16]. However, the treatment of IMOS is not standardized. Further analysis is mandatory. We considered that the role of open biopsy was not only for sampling but also for medullary decompression. In case 1, the pain remained but improved to a level controlled by NSAID administration after the bone hole was closed. In case 2, the pain has not recurred in the long follow-up duration after the bone hole was closed. In case 3, severe pain uncontrolled by even opioid use improved immediately after the open biopsy, although the patient kept taking the NSAIDs. The patient was able to discontinue the opioids. Thus, the open biopsy could be expected to reduce the severe pain in IMOS. Although a good result has been obtained during the period of our cases (39 months, 33 months, and 6 months), further analysis, with a longer follow-up period, is mandatory to determine whether the additional surgical treatment will be required.

In conclusion, 1. Massive sclerotic lesions with severe pain in the diaphyseal region of long bones should be considered as IMOS to avoid the delayed diagnosis, although other sclerotic bony lesions should be carefully excluded. 2. Triphasic bone scan with a fusiform-shaped intense area of tracer uptake on delayed bone image and without or slightly increased vascularity on blood pool image will help confirm IMOS. 3. The role of open biopsy with curettage as a minimally invasive procedure seemed to confirm the IMOS and pain relief in three cases, although more cases and long-term follow-up are necessary.

## Data Availability

To protect privacy and respect confidentiality, no raw data have been made available in any public repository. The original operation reports, intraoperative photographs, imaging studies, and outpatient clinic records are retained as per the normal procedure within the medical records of our institution. The datasets used and/or analyzed during the current study are available from the corresponding author on reasonable request.

## Abbreviations

IMOS: Intramedullary osteosclerosis
NSAIDs: Nonsteroidal anti-inflammatory drugs
CT: Computed tomography
MRI: Magnetic resonance imaging
SE: Spin-echo
TE: Echo time
TR: Repetition time
STIR: Short inversion time inversion recovery
MDP: Methylene diphosphonate
